# Mortality rate for mesothelioma as a basic cause in the period 2017–2022 in Chile

**DOI:** 10.3332/ecancer.2025.1927

**Published:** 2025-06-18

**Authors:** Francisca Quintanilla, Álvaro Torres, Monserrat Villar, Catalina Muñoz, José Lizama, Bárbara Mena

**Affiliations:** 1 School of Medicine, Pontificia Universidad Católica de Chile, Santiago 8330024, Chile; 2 School of Medicine, Universidad de Valparaíso, San Felipe 2170000, Chile; 3 School of Medicine, Universidad Mayor, Temuco 4810300, Chile; 4 School of Medicine, Universidad Andrés Bello, Santiago 8370146, Chile

**Keywords:** asbestos, Chile, mesothelioma, mortality, epidemiology

## Abstract

**Introduction:**

Mesothelioma is a neoplasm associated with asbestos exposure. Due to the lack of updated national epidemiological data, it was decided to determine the mortality rate (MT) due to mesothelioma during the period 2017–2022 in Chile.

**Materials and methods:**

Observational, descriptive and cross-sectional study. A descriptive analysis of mortality rate (TM) due to mesothelioma as a basic cause during the period 2017–2022 in Chile in patients aged 45 years and older was performed, analysing the variables sex, age, national region, anatomical site affected and place of death. Data were obtained from the Department of Health Statistics and Information and the National Institute of Statistics and were analysed using Microsoft Office Excel. No evaluation by an ethics committee was required.

**Results:**

We studied 362 deaths, determining a TM for the period of 0.97 deaths per 100,000 inhabitants, where the highest rate was recorded in 2019. There was a predominance in men, with a male excess MT of 2.19, as well as in those over 80 years of age and residents in the region of Arica and Parinacota. In most cases, the tumour location was not determined, but among those identified, the most frequent was of pleural origin. Most deaths occurred at home.

**Discussion:**

The findings show that the highest MTs are concentrated in men, the elderly and mining regions, groups in which exposure to asbestos is quite probable, both for occupational and residential reasons.

**Conclusion:**

The need to maintain active surveillance and high suspicion for mesothelioma is emphasised, with the application of public health measures for early diagnosis and adequate management.

## Introduction

Mesothelioma is a primary mesothelial cell neoplasm, with pleural origin being the most frequent location (65%–70%), followed by peritoneum (30%) and pericardium (1%–2%) [1, 2]. This neoplasm predominates in the male sex, with a p ico incidence between 50 and 60 years of age; moreover, it presents a higher prevalence in industrialised countries [2–4].

In the literature, most updated data pertain to the United States, United Kingdom and other industrialised countries in Europe, with a deficit of information regarding the remaining world population [3, 5]. Approximately 72% of European countries have now banned asbestos. The European Union implemented a total ban in 2005, although some countries, such as the United Kingdom, had already started to ban asbestos in the 1980s and 1990s, constituting the first places where bans on this type of material were implemented [6]. However, despite the bans, the risk persists due to the long latency of asbestos-related diseases, which can manifest decades after exposure [7, 8].

In terms of pathogenesis, the main cause of mesothelioma is exposure to asbestos, also known as asbestos in Spain and other regions of Europe; the small fibers of this material are inhaled and deposited in the lungs, triggering a chronic inflammatory response, cell damage and a wide range of mutations that eventually promote the development of cancer cells in the mesothelium [1, 3]. It is proposed that in the case of peritoneal mesothelioma, the fibers would be transported from the lungs via the lymphatic route [6, 9]. The latency period previously described reaches 30 to 50 years after exposure to asbestos, which makes early diagnosis difficult and makes it more prevalent in the elderly population [7].

In addition to asbestos exposure, other risk factors described are a genetic predisposition for germline mutations (particularly BRCA) and syndromes such as BAP-1, Lynch and Li-Fraumeni [1]. Other less described causes include exposure to carcinogens such as industrial chemicals, ionising radiation and radiotherapy [1, 10].

Clinical manifestations vary according to the location, but mainly include dyspnoea, cough, chest pain, fever, weight loss and night sweats, the main complication being pleural effusion refractory to adequate management. In the case of affecting the peritoneum, it is also associated with abdominal pain, abdominal distension, anorexia and ascites [1, 3, 5].

Diagnosis requires the integration of patient history, clinical presentation, laboratory and imaging; on the other hand, diagnostic confirmation is always performed by biopsy [3, 4]. To determine the characteristics of the tumour, lymphatic involvement and dissemination, computed tomography or magnetic resonance imaging should be performed; the latter is of choice in resectable cases; Positron emission tomography–computed tomography is also useful to detect dissemination [3].

Regarding treatment, multimodality therapy, which combines surgery, chemotherapy and radiotherapy, is the treatment of choice in the early stages of the disease [4, 11, 12]. However, most diagnoses are in advanced stages where the tumours are inoperable, being necessary other alternatives such as immunotherapy. The combination of Nivolumab and Ipilimumab has become the new standard of treatment for systemic approaches to advanced-stage malignant pleural mesothelioma, especially in cases where the disease has progressed after first-line chemotherapy [3, 13, 14].

In general, the prognosis is unfavourable. Five-year survival for pleural mesothelioma is only 5%, while for peritoneal mesothelioma it is 40% [4, 15]. The main causes of death described are pneumonia and embolisms; to a lesser extent, cardiac tamponade and invasion of large vessels [5].

Given the above, it is crucial to promote the study of mesothelioma, both from an epidemiological perspective to collect data on incidence and mortality, as well as through a more exhaustive analysis of its potential causal factors and therapeutic alternatives. This will make it possible to establish relevant public health policies, improve early detection, increase survival rates and raise the standard of living of those affected.

### Objectives

#### Main objective

To determine the mortality rate (MT) due to mesothelioma as a basic cause during the period 2017–2022 in Chile.

#### Secondary objectives

- Calculate the MT by sex.
- To describe the MT in different age groups.
- Define the MT in each national region.
- Estimate the percentage distribution (PD) of deaths by national region.
- To establish the PD by tumour location.
- To compare the PD of deaths by place of death.

## Materials and methods

Observational, descriptive and cross-sectional study, which analyses the MT due to mesothelioma as a basic cause in the population from 45 years of age in the period 2017–2022 in Chile. The following variables were examined: sex, age, region, anatomical site affected and place of death. Data were extracted from the databases of the Chilean Department of Health Statistics and Information (DEIS) and the National Institute of Statistics (INE).

A descriptive data analysis was performed, using measures of central tendency such as average and percentages, and TMs were calculated, followed by tabulation and creation of graphs. Microsoft Office Excel was used for data processing. Two formulas were applied to analyse mortality in the population:

*MT: Number of deaths due to Mesothelioma in a given year total population at risk x 100.000*

*Over MT according to sex: MM according to male sex MM according to female sex*

No evaluation by an ethics committee was required because the data used came from publicly available sources, and the personal information of each patient surveyed was duly encrypted prior to publication by the DEIS and the INE in order to preserve confidentiality. The authors declare that they have no conflicts of interest.

## Results

A total of 362 deaths due to mesothelioma as a basic cause were studied in the period 2017–2022 in patients aged 45 years and older, thus determining a TM for the period between the aforementioned years of 0.97 deaths per 100,000 population. During 2017, a mesothelioma MT of 0.96 deaths per 100,000 inhabitants was recorded; the following year, there was a decrease to 0.72 deaths per 100,000 inhabitants, this being the lowest figure during the period studied. In 2019, the highest rate was observed with 1.22 deaths per 100,000 population, then during 2020, a rate of 1.06 was recorded. The 2021 continued to show a decreasing trend with a rate of 0.91 deaths per 100,000 inhabitants, and finally, in 2022 continued with a similar trend with a rate of 0.93 deaths per 100,000 inhabitants.

**Figure 1.**
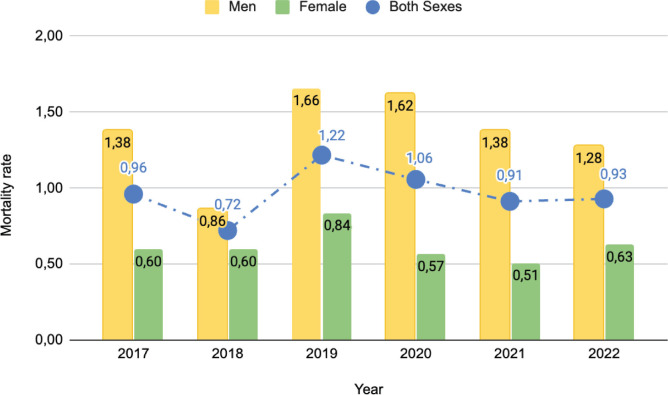
MT in Chile from age 45 by sex, period 2017–2022.

During the period studied, it was observed that men had a higher MT due to mesothelioma than women, with 1.36 deaths per 100,000 inhabitants for men and 0.63 for women. Thus, determining a male excess MT in the period studied of 2.19. It is noteworthy that both sexes had their highest MTs in 2019, with 1.66 deaths per 100,000 inhabitants for men and 0.84 for women. The lowest rates were recorded in 2018, with 0.86 for men and in 2021, with 0.51 deaths per 100,000 inhabitants for women. During the entire period studied, the male TM was consistently higher than the female TM (Figure 1).

MT in Chile from age 45 by sex, period 2017–2022.

The population was divided into three age groups (45 to 64 years, 65 to 79 years, and 80 years and older), where the highest MT was found in the 80 years and older age group in all the years studied, resulting in a TM of 2.51 deaths per 100,000 inhabitants for the period. On the other hand, the group aged 45 to 64 years had the lowest MT during the entire period studied, with 0.44 deaths per 100,000 inhabitants (Table 1).

**Figure 2.**
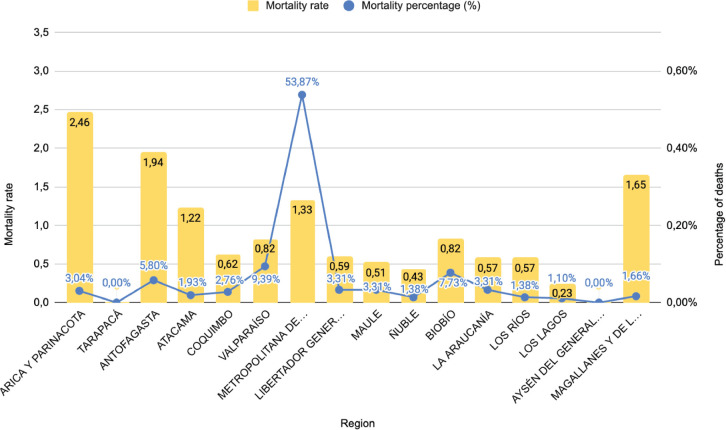
MT and percentage of mesothelioma deaths by Chilean region since the age of 45 years, period 2017–2022.

By national region, the highest TM for the period was recorded in the region of Arica and Parinacota with 2.46 deaths per 100,000 inhabitants, followed by the region of Antofagasta with 1.94 and Magallanes in third place with 1.65 deaths per 100,000 inhabitants. On the other hand, in both the Tarapacá and Aysén regions, no deaths from mesothelioma were recorded in the population over 45 years of age during the period studied (Figure 2). Regarding the PD of deaths by national region, it is noteworthy that the Metropolitan region presents a large national proportion with 53.87% (*n* = 195) of the cases registered in the period, while the rest of the regions maintained a homogeneous PD, with the second majority being the Valparaíso region with 9.39% (*n* = 34). On the other hand, the region with the lowest percentage was Los Lagos with 1.10% (*n* = 1) of the total cases, excluding the regions of Tarapacá and Aysén where no deaths were recorded (Figure 2).

TM and % mesothelioma deaths by Chilean region from age 45 years, period 2017–2022.

**Figure 3.**
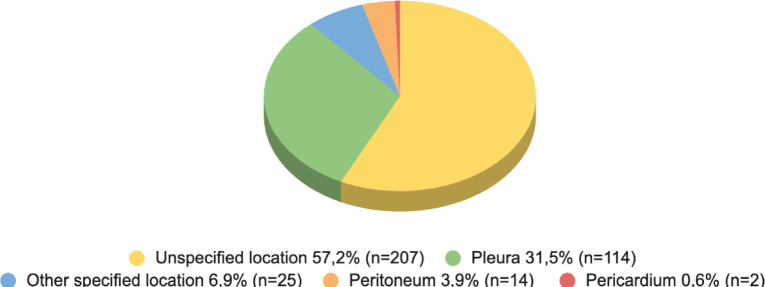
Deaths due to mesothelioma according to tumour location in Chile since the age of 45 years, period 2017–2022.

According to tumour location in the period, the highest number of deaths belonged to mesothelioma of unspecified site with 57.2% (*n* = 207) of deaths, followed by mesothelioma of the pleura with 31.5% (*n* = 114) (Figure 3).

Deaths from mesothelioma according to tumour location in Chile since the age of 45, period 2017–2022.

According to the place of death, during the period studied, the highest number of deaths occurred at home with 62.14% (*n* = 225) of deaths, followed by clinics or hospitals with 37.29% (*n* = 135) of deaths and the lowest number occurred in other places with 0.55% (*n* = 2).

## Discussion

Throughout the period studied, the mesothelioma MT behaved in a variable manner over the years, without showing a trend that demonstrates a progressive increase or decrease over time. However, during 2020 and 2021, there was a decrease in the MT compared to 2019, which may be associated with an increase in infection and death due to SARS-CoV-2, making it the leading cause of death [16, 17].

Regarding the MT according to sex, the male MT is consistently higher than the female MT, which is associated with the fact that occupational exposure to asbestos occurs in work contexts that include construction, manufacturing and other industrial environments, which tend to have mainly male workers [18]. In addition, it has been shown that, in situations where other negative prognostic factors are not present, women with a specific type of mesothelioma (epithelioid) show a survival advantage compared to men, as well as a higher survival rate after surgery [19], which could also explain the higher mortality in males.

Regarding age groups, it is observed that the increase in age is related to an increase in the MT, which can be attributed to both physiological conditions typical of advancing age and chronic pathological conditions of adults and older adults, which generate a deterioration in the general state of health and in the response capacity of the organism. On the other hand, it should be considered that the latency period, which is the period from asbestos exposure to the expression of the disease, is approximately 40 years [7], so it is to be expected that the MT from mesothelioma is higher in people of older ages considering that exposure to asbestos was predominantly at working ages.

At the national level, the highest MTs are found mainly in the northern regions, with the highest rates in Arica and Parinacota and Antofagasta, which is consistent with the fact that the north of the country is characterised as a mining area, with up to 20% of its population working in this area [20] and, therefore, is at risk of continuous exposure to asbestos and of suffering subsequent complications such as mesothelioma. On the other hand, the fact that the Metropolitan, Valparaíso and Biobío regions have the highest PD of deaths can be explained by the fact that these regions have a greater number of medical specialists who are able to make an accurate diagnosis of the pathology, especially the Metropolitan region which, in 2015, had 40% of the medical specialists in Chile, followed by the Valparaíso and Bio Bío regions with 10% and 14%, respectively [21], which is consistent with the data obtained in our study.

Regarding the tumour location, most of the mesothelioma cases recorded did not have a specified body site, this could be attributed to the fact that in medical practice it usually happens that at the beginning of the diagnostic process and study the pathology is entered into the system as an unspecified body site and subsequently it is not changed. Another explanation would be the technical complexity and high costs to perform the diagnosis of the primary tumour, since many centers do not have these resources, in addition to the high risk and difficulty involved in invasive procedures in oncological patients or older adults, which could limit the number of studies that can be performed [22, 23]. On the other hand, the second most frequent location is the pleural, which is consistent with world epidemiology in being the most frequent specific location and is due to the high exposure of the respiratory system to environmental pollutants, including asbestos, while the peritoneum and pericardium are organs that are less exposed to carcinogens, which explains why they have the lowest PDs [1, 3].

The largest number of deaths occurred at home or in the home, which can be attributed to the fact that these patients are generally referred to palliative care for imminent end of life, in this context patients prefer to be at home with their families rather than in a hospital, while on the other hand, it could be attributed to a deficiency of medical care that did not allow certain patients a timely diagnosis and proper subsequent management [24].

The data presented were extracted by the DEIS of Chile, which guarantees their representativeness at the national level, thus granting reliability and relevance to our findings. On the other hand, it is important to mention the existing limitation regarding the availability of information on the subject at both the global and national levels, which makes it difficult to obtain, compare and contrast data.

This study has limitations that should be considered. First, a greater analysis of possible etiologies could have been achieved if data on the occupational exposures of patients with mesothelioma were available, which would have made it easier to identify the primary causal factor. Second, both the DEIS and the INE only provide data on population deaths, without providing individual clinical information on each patient, which limits knowledge on the treatments administered and the possible correlations between outcomes and the interventions received.

In the context of mesothelioma, the study establishes a basis that could be useful for future research. It is essential to further explore the occupational characteristics and work histories of patients, which would elucidate patterns of asbestos exposure and associated risk factors more precisely. It would also be relevant to investigate biological differences between sexes that could explain variations in survival and responses to treatment, which would open up the possibility of personalised therapies. At the regional level, the development of studies that analyse the differences in the accessibility and quality of diagnosis in different areas of the country would make it possible to identify critical gaps in the health system and design targeted interventions. Finally, given the environmental and occupational impact of asbestos, future research could focus on evaluating current public policies, as well as promoting primary and secondary prevention strategies to reduce the incidence of mesothelioma. These lines of research would contribute not only to the advancement of knowledge about this disease, but also to the strengthening of public health policies and the well-being of the most exposed communities.

## Conclusion

Mesothelioma is a primary neoplasm that persists today, even in spite of the numerous prohibitive strategies implemented by several countries. This work provides information in a field that has been little explored both nationally and internationally, which will give greater visibility to this pathology, in addition to providing a perspective of it at the country level that can be used both for further studies and to generate effective health measures. Due to the high association with certain work activities, it is relevant that in medical practice, a detailed clinical history is taken and that there is a high level of suspicion, in addition to which it is imperative to homogenise the number of medical specialists in regions to achieve timely care and diagnosis.

## Conflicts of interest

The authors declare that they have no conflicts of interest.

## Funding

This study was supported by ecancer (UK Charity number 1176307), which covered publication costs.

## Figures and Tables

**Table 1.** MT in Chile from age 45 by age intervals, period 2017–2022.

	2017	2018	2019	2020	2021	2022	Period
45–64	0.42	0.47	0.47	0.45	0.47	0.38	0.44
65–79	1.89	1.24	2.68	2.28	1.57	1.96	1.94
80 and over	2.76	1.27	3.19	2.55	2.76	2.55	2.51
